# The anthropometry of children and adolescents may be influenced by the prenatal smoking habits of their grandmothers: A longitudinal cohort study

**DOI:** 10.1002/ajhb.22594

**Published:** 2014-08-18

**Authors:** Jean Golding, Kate Northstone, Steven Gregory, Laura L Miller, Marcus Pembrey

**Affiliations:** Centre for Child and Adolescent Health, School of Social and Community Medicine, University of BristolBristol, BS8 2BN, United Kingdom

## Abstract

**Objectives:**

Previously, in the Avon Longitudinal Study of Parents and Children (ALSPAC), we have shown different sex-specific birth anthropometric measurements contingent upon whether or not prenatal smoking was undertaken by paternal grandmother (PGM±), maternal grandmother (MGM±), and the study mother (M±). The findings raised the question as to whether there were long-term associations on the growth of the study children over time.

**Methods:**

Measures of weight, height, body mass index, waist circumference, lean mass, and fat mass of children in the ALSPAC study from 7 to 17 years of age were used. We compared growth in four categories at each age: PGM+M− with PGM−M−; MGM+M− with MGM−M−; PGM+M+ with PGM−M+; MGM+M+ with MGM−M+; and adjusted for housing tenure, maternal education, parity, and paternal smoking at the start of the study pregnancy.

**Results:**

We found that if the PGM had, but the study mother had not, smoked in pregnancy, the girls were taller and both genders had greater bone and lean mass. However, if the MGM had smoked prenatally but the mother had not (MGM+M−), the boys became heavier than expected with increasing age—an association that was particularly due to lean rather than fat mass, reflected in increased strength and fitness. When both the maternal grandmother and the mother had smoked (MGM+M+) girls had reduced height, weight, and fat/lean/bone mass when compared with girls born to smoking mothers whose own mothers had not smoked (MGM−M+).

**Conclusions:**

This study indicates that smoking in humans can have sex-specific transgenerational effects. Am. J. Hum. Biol. 26:731–739, 2014. © 2014 The Authors American Journal of Human Biology Published by Wiley Periodicals, Inc.

Our program of research into transgenerational effects of cigarette smoking (Miller et al., 2014; Northstone et al., 2014) was instigated as a result of studies from Sweden. These were based on samples of individuals born close to the Arctic Circle in the town of Överkalix. Their longevity and other health outcomes were linked to detailed historical records of harvests experienced by their ancestors (Bygren et al., 2001). Using three independent birth cohorts in the years 1890, 1905, and 1920, Kaati et al. (2002) showed that the paternal grandfathers' plentiful food supply in mid-childhood was associated with a fourfold increased chance of diabetes on the grandchild's death certificate [95% CI 1.3, 12.9]. Their study also showed that cardiovascular mortality in the study individuals was reduced when there had been poor food supply in the father's mid-childhood. Subsequently, sex-specific analysis of the data showed that the mortality rate of the men born in the target years was linked to their paternal grandfather's food supply in mid-childhood, whereas the mortality rate of the women studied was associated solely with their paternal grandmother's food supply (Pembrey et al., 2006). This association was shown in two of three independent cohorts. Exposure sensitive periods involved both paternal grandparents' mid-childhood but also the fetal/infant period for the paternal grandmothers.

In the UK, since the Second World War, there have been no particular years of starvation or glut. In the search for an environmental feature that we could time in regard to the age of exposure at which it occurred, we have chosen the smoking habits of the individual parents. It is well recognized that smoking has strong effects on various physiological systems, and results in a loss of appetite and general reduction in weight compared with nonsmokers (Chiolero et al., 2008). Previously, using the Avon Longitudinal Study of Parents and Children (ALSPAC), we have shown that fathers who started smoking regularly between the ages of 8 and 11 had boys (but not girls) with increased body mass index (BMI), waist circumference, and body fat mass as teenagers (Northstone et al., 2014). We have also shown that nonsmoking mothers exposed prenatally to their own mothers' smoking delivered children who were larger at birth (Miller et al., 2014). After adjustment, the average birth weight, birth length, and BMI measurements of the boys (but not the girls) were greater if the maternal grandmother smoked prenatally: birth weight = +61 [95% CI +30, +92] g; birth length = +0·19 [95% CI +0·02, +0·35] cm; birth BMI = +1·6 [95% CI +0·6, +2·6] g/m^2^. In a parallel paper (Pembrey et al., 2014), we have shown that exposure of the father to his mother's smoking resulted in a reduction in birth head circumference of his sons if the study mother also smoked in pregnancy, and that this was reflected in reduced IQ in this group. Here, we examine the growth of these children from ages 7 to 17 to determine whether prenatal smoke exposure of either parent is associated with the growth of the offspring, including body composition, and whether it is sex-specific and/or depends on whether the study mother smoked in pregnancy.

## MATERIALS AND METHODS

### Study samples

The data used in these analyses were collected as part of the ALSPAC, which was designed to assess the ways in which the environment interacts with the genotype to influence health and development (Golding, 2004). Pregnant women resident in the study area in south-west England with an expected date of delivery between 1st April, 1991 and 31st December, 1992 were invited to take part. About 80% of the eligible population did so (Boyd et al., 2013). The initial ALSPAC sample consisted of 14,541 pregnancies; of these, 14,472 had known birth outcomes: 14,062 were live births and 13,988 were alive at 1 year.

**Figure 1:**
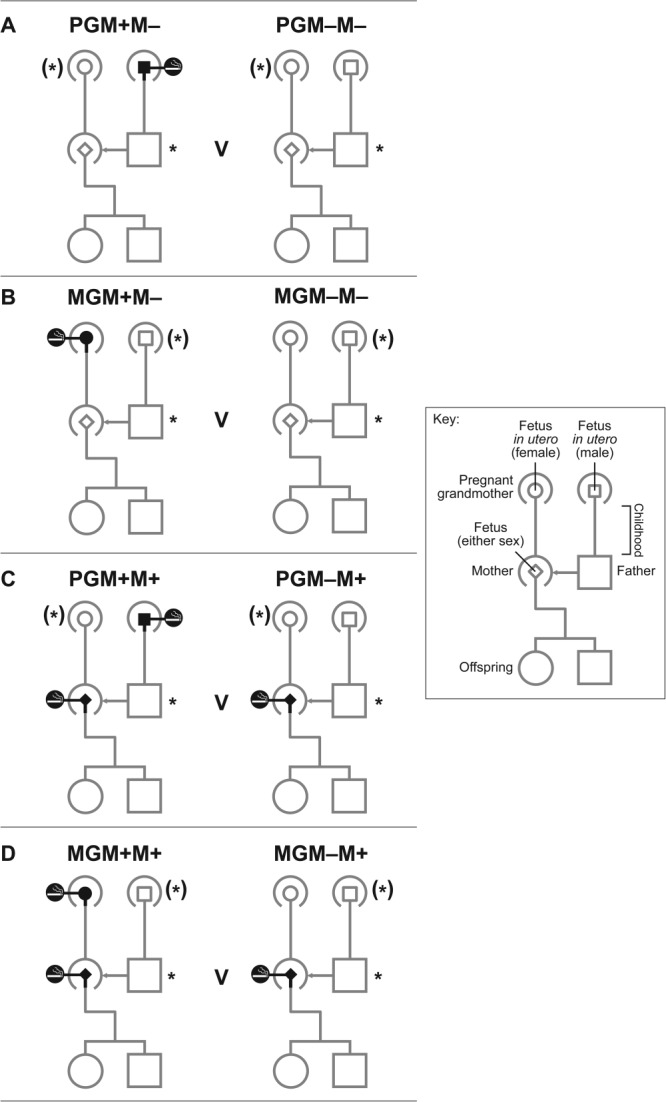
Developmental pedigrees illustrating the prenatal smoke exposures studied. (*all analyses adjusted for paternal smoking; (*) smoking of other grandmother adjusted for in sensitivity analysis)

Information collected from the parents during their study pregnancy included details of the maternal and paternal grandparents. The two pathways of possible influence of parental prenatal exposure to cigarette smoke on the study child that we will investigate in this article, concern (a) via the maternal grandmother (MGM) to the mother (M) in utero to her study fetus, and (b) via the paternal grandmother (PGM) to the study father (F) while he was in utero and thence to the study conceptus (Fig. 1).

### The exposures

The pregnant study mothers and their partners were sent six questionnaires during pregnancy (ALSPAC, 2014). These elicited information on their current smoking habits and those of their parents (i.e., the study grandparents). If they reported that their mothers had smoked, they were asked whether their mothers had smoked when expecting them—and, if so, were given the responses yes/no/do not know from which to select. Thus, the parents who replied “do not know,” had a mother who smoked but the parent was unsure whether she had smoked during her pregnancy. We have analyzed these data assuming that these women did smoke during pregnancy.

### Possible confounders

Potential confounders included in the analyses were the study mother's parity (as ascertained from the maternal report of previous pregnancies resulting in either a live- or still-birth, and coded as 0; 1+); mother's partner smoking during the pregnancy (primarily reported by partner, but maternal report was used if partner report was missing: yes; no); housing tenure as a measure of socioeconomic background (owned or mortgaged; rented public housing; all other), and maternal education (highest level of educational attainment—in five levels of increasing achievement).

### Outcomes

Children were measured using standardized methods by the ALSPAC study team in a clinic setting from the age of 7 and every other year thereafter until the age of 17.

Height was measured using the Harpenden Stadiometer (Crymych, UK): shoes were removed, the study child stood with feet flat, so that the under-side of the heels was in contact with the ground. The heels were placed together, so that the medial malleoli were touching (unless the child had knock knees). The child stood straight so that heels, calves, buttocks, and shoulders were in contact with the vertical backboard of the Stadiometer. Shoulders were relaxed and sloping forward in a natural position, hands and arms were loose and relaxed with palms facing medially. The headboard was slid down the backboard until it touched the study child's head. To ensure that the head stayed in contact with the headboard and to minimize the effect of hair thickness, a 1 kg weight was placed on the headboard. The height was recorded to the last completed millimeter.

Waist circumference was measured as the minimum circumference of the abdomen between the iliac crests and the lowest ribs, with the tape perpendicular to the long axis of the body touching the skin but not compressing the tissue. It was measured to the last complete millimeter.

Weight was measured using Tanita scales Body Fat Analyzer model TBF 305 (Arlington Heights, IL). The child was encouraged to pass urine and undress to their underclothes. BMI was calculated as weight (kg) (height (m))^2^. Total body fat, lean, and bone mass were measured bi-annually from the age of 9 using total-body dual-energy X-ray absorptiometry scans, performed using a Lunar Prodigy dual-energy X-ray absorptiometer (GE Medical Systems Lunar, Madison, WI) (Toschke et al., 2007). Bone mass, lean mass, and fat mass were estimated at each age.

Grip strength was assessed at age 11 using the Jamar hand dynamometer, which measures isometric strength in kilograms. The child sat in a chair with arms and back support and was asked to rest his/her forearms on the arms of the chair with their wrist just over the end of the arm of the chair. The wrist was placed in a neutral position with the thumb facing upwards. The tester demonstrated how to use the dynamometer to the child showing how gripping very tightly registered the best score. The child was given a practice squeeze of the dynamometer to ensure that it felt comfortable. Starting with the right hand, the hand was positioned so that the thumb was round one side of the handle and the four fingers were around the other side. It was important that the instrument felt comfortable for the child and the position of the handle was altered if necessary. The measurer rested the base of the dynamometer on the palm of the child's hand in order to support the weight of the dynamometer, whilst ensuring that the movement of the machine was not restricted. The child was encourage to squeeze as long and as tightly as possible or until the needle stopped rising: the higher the reading, the stronger the grip. The grip strength was measured twice in each hand and the mean of the 4 measurements was used.

### Cardio-respiratory fitness

Physical work capacity (Watts) was assessed at a heart rate of 170 bpm (PWC_170_). This was estimated using standard regression methods from parameters measured using an electronically braked cycle ergometer (Lawlor et al., 2008).

### Statistical analyses

Multivariable linear regression models assessed the grandchildren's mean height, weight, BMI, waist circumference, fat mass, lean mass, and bone mass in regard to the parental prenatal smoking exposures. All models were adjusted for parity, maternal education, paternal smoking at the start of pregnancy, and housing tenure. Because maternal prenatal smoking itself is associated with overweight in the offspring (Oken et al., 2007; Ino, 2010), we have analyzed separately the children whose mothers themselves smoked during pregnancy. In line with the evidence in the literature that various effects of cigarette smoking are sex specific (e.g., Zaren et al., 2000), together with the results from our earlier studies (Miller et al., 2014; Northstone et al., 2014; Pembrey et al., 2006), we have analyzed the male and female offspring separately and, where appropriate, have tested for interactions with sex.

## RESULTS

### Response

The numbers of study children attending for examination at each of the time-points are shown in Table1. It can be seen that there is a steady decline in attendance from 8,290 at age 7 to 5,217 at age 17. However, there was no bias over time in the proportion of the children attending for whom data were available on grandmaternal prenatal smoking—this varied from 88.2% to 89.5% for the MGM history, and from 72.3% to 74.3% for that of the PGM.

**Table 1:** Attendance at the clinics at which anthropometric measurements were made, and proportions with data on the smoking of the maternal and paternal grandmothers in utero

Age at focus clinic	No. attending	No.(%) with information on MGM	No. (%) with information on PGM
7 Years	8,290	7,352 (88.7%)	5,994 (72.3%)
9 Years	7,722	6,869 (89.0%)	5,602 (72.5%)
11 Years	7,153	6,395 (89.4%)	5,221 (73.0%)
13 Years	6,147	5,504 (89.5%)	4,544 (73.9%)
15 Years	5,515	4,931 (89.4%)	4,096 (74.3%)
17 Years	5,217	4,601 (88.2%)	3,795 (72.7%)

MGM, maternal grandmother; PGM, paternal grandmother.

### Anthropometric measures

The results of comparing the anthropometric measures between the children whose grandmothers had smoked while one of their parents was in utero are shown in Supporting Information Tables 1–4 and summarized below.

### Child's height

There were no apparent differences in height associated with the mother's prenatal exposure, unless she smoked herself. In the latter scenario [MGM+M+ vs. MGM−M+], her girls were consistently of lower height than expected, ranging from 0.9 to 1.8 cm lower (Supporting Information Table 4).

In contrast, there was a consistency in regard to the father's prenatal exposure—the study children, especially the girls, were taller than expected provided their own mother did not smoke [PGM+M−]. The excess adjusted height varied from 0.2 to 0.7 cm for boys and 0.4 to 0.7 cm for girls (Supporting Information Table 1).

### Child's weight, BMI, and waist circumference

**Figure 2:**
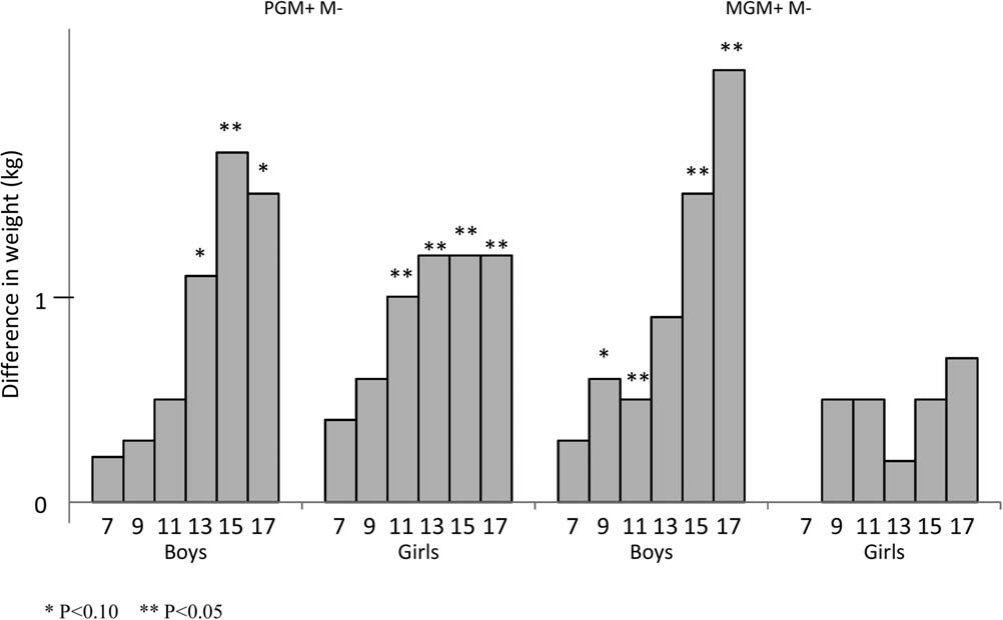
Weight of offspring of nonsmoking women showing the difference (kg) between those whose grandmothers smoked prenatally compared with those who did not (MGM, maternal grandmother; PGM, paternal grandmother; M, mother; +, smoked prenatally; −, did not smoke prenatally).

There were interesting differences in weight in the children of the nonsmoking mother according to whether she or her partner was exposed in utero. The adjusted differences are shown in Figure 2. The increased child weight with the maternal grandmother smoking [MGM+M− vs. MGM−M−] was apparent for just the boys, whereas that with the paternal grandmother smoking when pregnant [PGM+M− vs. PGM−M] resulted in increased weight in both boys and girls during adolescence (Supporting Information Tables 1 and 2).

Similarly among nonsmoking women, there were positive associations with BMI which increased with age among both genders for the paternal grandmother smoking in pregnancy but similar effects were much stronger among the boys rather than the girls when the maternal grandmother had smoked. For waist circumference, there were increases in offspring of nonsmoking women if either grandmother had smoked, but the effects were slightly stronger in boys when the MGM had smoked and in girls when the PGM had smoked.

If the mother herself had smoked prenatally, there was no discernible effect of the paternal grandmother smoking prenatally [PGM+M+ vs. PGM−M+] on the weight, BMI, or waist circumference of the child. However, if the maternal grandmother had smoked in pregnancy [MGM+M+ vs. MGM−M+], the study girls [but not boys] tended to weigh less [ranging from 0.9 to 2.2 kg] have slightly lower BMIs and reduced waist circumference [ranging from 0 to 1.7 cm].

### Child's components of body composition

**Figure 3:**
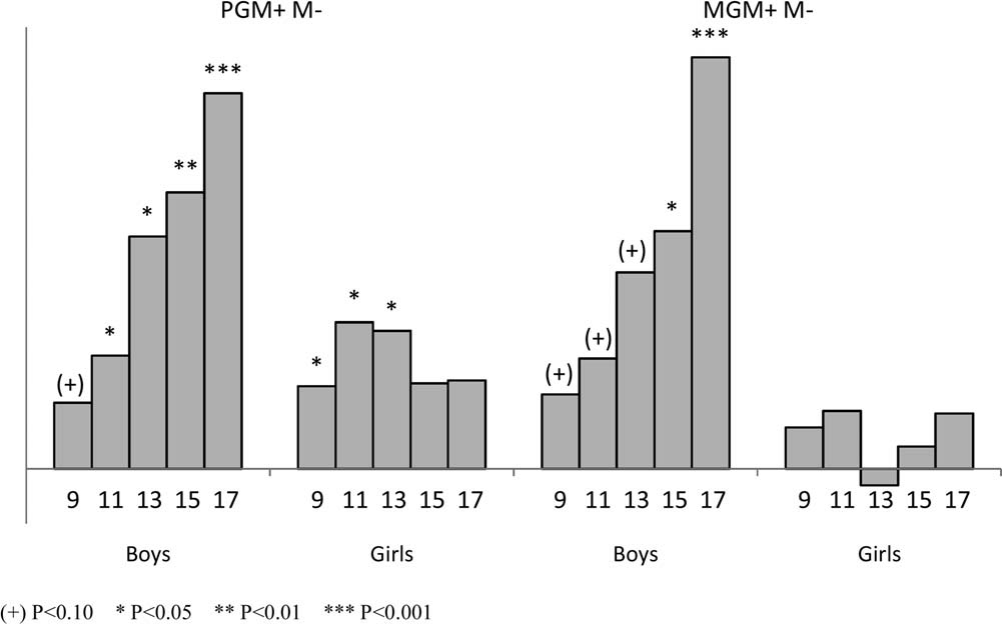
Lean mass of offspring of nonsmoking women showing the difference between those whose grandmothers smoked prenatally compared with those who did not (MGM, maternal grandmother; PGM, paternal grandmother; M, mother; +, smoked prenatally; −, did not smoke prenatally).

For children of mothers who did not smoke prenatally, the effect of the paternal grandmother smoking in pregnancy [PGM+M− vs. PGM−M−] indicated a slightly increased fat mass in the girls [ranging from 0.30 to 0.75 kg], increases in bone mass in both sexes, and strong effects on lean mass that increased with age for the boys, but was less striking for the grand-daughters after 13 years of age. By age 17, the difference between the sexes was significant [interaction *P* = 0.012]. If the maternal grandmother had smoked prenatally and her daughter had not [MGM+M− vs. MGM−M−], there was little effect on the child's fat mass or bone mass, but there was a strong positive association with lean mass in the study boys, but not the girls (*P* for interaction at age 17 = 0.006; Fig. 3).

If the study mother had smoked prenatally, there were no consistent associations between the child's body composition with the history of the paternal grandmother's smoking [PGM+M+ vs. PGM−M+], but some indication that if the maternal grandmother had smoked prenatally [MGM+M+ vs. MGM−M+] the girls had slightly lower fat, lean, and bone mass than expected from the factors taken into account. The difference between the genders was significant for both lean mass and bone mass at age 9 (*P* = 0.003 and 0.045, respectively).

### Strength and fitness

Given the unexpected associations with lean mass, we carried out further analyses to determine whether the increase in lean mass was reflected in an increase in strength and/or fitness. We therefore looked at the mean levels of these outcomes using the same comparisons and confounders as for the anthropometry measures. The results are shown in Supporting Information Table 5. In brief, there was an association if the maternal grandmother had smoked prenatally but the study mother had not [MGM+M− vs. MGM−M−], with an increase in grip strength in the boys +0.52 [95% CI +0.11, +0.92; *P* = 0.012], but not girls −0.18 [95% CI −0.56, +0.20]; the test for interaction between the sexes gave *P* = 0.10. For fitness, there was also a positive effect for this group of boys +2.08 [95% CI +0.91, +3.26; *P* = 0.001] but not girls: +0.38 [95% CI −0.74, +1.50; *P* = 0.503]; test for interaction *P* = 0.061.

### Multiple testing

This set of analyses has been designed to look at ways in which the grandmothers' prenatal smoking has influenced the growth of the study child. It is hypothesis generating. There are no other studies to our knowledge that can be used to attempt to replicate our results at this point in time. We are therefore reluctant to be too astringent in rejecting results that do not reach either a Bonferroni or other test for multiple testing. We therefore deliberately take a basic approach, and assess the numbers of results with *P* values <0.10 or less. These are shown in Table2 for each anthropometric measure.

**Table 2:** The number of adjusted associations with P < 0.10 (P < 0.05) for each measurement, exposure category, and sex of the study child

	PGM+M−		PGM+M+		MGM+M−		MGM+M+	
Boy	Girl	Boy	Girl	Boy	Girl	Boy	Girl
Height	0	5 (3)	0	0	0	0	0	2 (1)
Weight	3 (1)	6 (4)	0	0	4 (3)	0	0	3 (2)
BMI	1 (1)	3 (1)	0	0	5 (5)	0	0	1 (0)
Waist circumference	0	4 (3)	0	1 (1)	4 (3)	1 (0)	0	1 (1)
Fat mass	5 (0)	3 (1)	0	0	1 (0)	0	0	1 (0)
Lean mass	5 (3)	3 (3)	0	0	5 (2)	0	0	1 (1)
Bone mass	5 (3)	4 (1)	0	0	1 (1)	0	0	0
Column total	19 (8)	28 (16)	0	1 (1)	20 (14)	1 (0)	0	9 (5)

MGM, maternal grandmother; PGM, paternal grandmother; M, mother; +, smoked prenatally; −, did not smoke prenatally. Expected numbers in each cell = 0.6 (0.3) for height, weight, and BMI; 0.5 (0.25) for the other measures; expected column totals = 3.8 (1.9).

A clear pattern appears—for each of the eight groups being compared there are 38 sets of analyses, and we would therefore expect 3.8 of these to have *P* < 0.10 and 1.9 with *P* < 0.05 by definition. There were just four of the eight groups that clearly showed associations in excess of this: the comparisons of PGM+M− with PGM−M− for both girls and boys; MGM+M− with MGM−M− for boys only, and MGM+M+ with MGM−M+ for girls (Table2). Not all associations are mutually exclusive but many are—e.g., MGM+M+ and MGM+M−.

## DISCUSSION

This study was designed to determine whether prenatal smoking by either grandmother had discernible effects on the growth of her child. We have compared seven different anthropometric measures at six time points, distinguishing between the sexes, and comparing four different groups: PGM+M− with PGM−M−; MGM+M− with MGM−M−; PGM+M+ with PGM−M+ and MGM+M+ with MGM−M+. The results are summarized in Table3, which also include birth measurements from our previous study (Miller et al., 2014; Pembrey et al., 2014). Epidemiological strategies often include the search for patterns (Wilson, 1994) and this is the strategy we have used in this set of analyses.

**Table 3:** Pattern of associations at birth and in childhood for each measurement, sex, and prenatal smoke exposure category

	PGM+M−		PGM+M+		MGM+M−		MGM+M+	
Boy	Girl	Boy	Girl	Boy	Girl	Boy	Girl
Birth								
Weight	·	·	·	·	↑	·	·	·
Length	·	·	·	·	↑	·	·	·
BMI	·	·	·	·	↑	·	·	·
Head circumference	·	·	↓	·	·	·	·	·
Childhood								
Height	·	↑	·	·	·	·	·	↓
Weight	↑	↑	·	·	↑	·	·	↓
BMI	↑	↑	·	·	↑	·	·	·
Waist circumference	·	↑	·	·	↑	(↑)	·	↓
Fat mass	·	↑	·	·	·	·	·	↓
Lean mass	↑↑	↑	·	·	↑↑	·	·	↓
Bone mass	↑	↑	·	·	·	·	·	↓

MGM, maternal grandmother; PGM, paternal grandmother; M, mother; +, smoked prenatally; −, did not smoke prenatally; **↑**, positive association; ·, no consistent association; **↓**, negative association.

Additional Supporting Information may be found in the online version of this article.

Supplementary Information

### Maternal grandmother

Our original studies had shown that there was an association of increased fetal growth (increased birth weight, birth length, and birth BMI) for boys but not girls when the maternal grandmothers smoked prenatally provided their mothers did not [MGM+M−] (Miller et al., 2014). One interpretation of these results, in line with the “predictive adaptive response” hypothesis (Gluckman et al., 2005; Godfrey et al., 2010) was that the mother was “primed” in utero to anticipate an environment which would reduce the growth of her own fetus, in consequence of which she “programmed” her fetus to grow faster than usual. Another way of looking at this maternal influence on fetal growth is that her ability to constrain the paternally driven fetal growth (e.g., via IGF2 (Demetriou et al., 2014)) was impaired by her own exposure to smoke in utero. This interpretation is more in line with the evolutionary conflict theory of sexually antagonistic traits (Frank and Crespi, 2011). The latter theory might also help to explain why this increased growth only appeared to apply to male fetuses.

In this study, we have shown that the boys with this history [MGM+M−] continued to have increased growth, particularly in regard to weight, BMI, and waist circumference. The associations were again not found with girls. Analyses to distinguish the components of the increase in weight in this group of boys showed that the increase was due to lean mass and not to fat mass or bone mass. Because lean but not fat mass has been shown to correlate well with cardio-respiratory fitness and with grip strength (Sherriff et al., 2009), we assessed whether the increase in lean mass found here was reflected in either strength or fitness, and found both an increase in grip strength and in fitness in this group of boys. There were no such associations in girls.

In regard to the maternal grandmothers who smoked in pregnancy, if their daughters also smoked prenatally, we found no unexpected associations with the growth of their boys, but their girls showed reduced height, weight, waist circumference, lean, fat, and bone mass. In line with the observation that, in contrast to the boys, the fetal growth of MGM+M− girls did not “overcompensate,” girls rather than boys show reduced growth when both grandmother and mother smoked in pregnancy compared with mother only smoking.

### Paternal grandmother

In our earlier studies, we showed that there was no discernible effect on birth weight, birth length, and birth BMI if the paternal grandmother had smoked prenatally. This was true whether or not the mother herself smoked prenatally (Pembrey et al., 2014). However, when the mother herself smoked in pregnancy, we did find a strong effect of smoking by the paternal grandmother on the head circumference of the boys (but not the girls). This finding was reflected in a reduction in IQ of this group of children.

In the present study of growth in childhood and adolescence, however, we show no associations comparing PGM+M+ with PGM−M+. In contrast, if the paternal grandmother had smoked prenatally but the study mother did not smoke [PGM+M−], there were indications of increased growth in both boys and girls. All components of growth appeared to be involved in girls, including height, waist circumference, and fat mass as well as lean and bone mass. The boys have increased weight, BMI, bone, and lean mass, like the boys with smoking maternal grandmothers; however, this was not reflected in increased strength or fitness.

### Strengths and weaknesses

These analyses are designed to assess whether a history of parental exposure in utero to smoking has discernible effects on the growth of their children. The study benefits from being based on a geographically defined population, and collecting information on grandparental and maternal smoking habits before the birth of the study child, thus being clear of any bias in knowing details of the child's growth. The disadvantage of the study is that there are no other human datasets currently available with which to test the hypotheses raised by this study. However, we hope that these results will prompt other longitudinal studies to be designed to collect data from as many generations as possible.

### Possible explanation

The aim of this study was to assess the impact (if any) of prenatal smoking of each grandmother on the growth of the study boys and girls up to age 17 years; and where differences were observed to note any particular patterns. A key intermediate variable in any transgenerational effect was whether or not the mother herself smoked in the pregnancy that gave rise to the child, as is clear from Table3. It is important to note that this study is primarily about the effect of the mother or father being exposed in utero so when the mother also smoked in pregnancy the comparison is MGM+M+ versus MGM−M+ (or PGM+M+ vs. PGM−M+) not MGM+M+ vs. MGM−M−. The broad conclusion from Table3 is that paternal exposure in utero is linked to increased growth in his children when his partner does not smoke. However, when his partner smokes in pregnancy, this has the effect of overriding these, possibly adaptive, gains in growth—there is no difference from the growth of children of smoking mothers. For maternal exposure in utero, there is a gain in growth (plus strength/fitness) but only for her sons, when she does not smoke herself. If she also smokes in pregnancy, her sons grow just like sons of smoking mothers, but her daughters have reduced growth below that of daughters of smoking mothers. Thus, it appears that the parental smoke exposure in utero sets a (sex) specific potential growth trajectory for their future children, but with maternal smoking in addition the child's growth is reduced by a particular amount from that trajectory.

Explaining the sex differences is a challenge: (i) in terms of whether there are any effects on adolescent growth or not, and (ii) in terms of the differences in individual anthropometric measures of boys and girls when there are transgenerational effects. As noted earlier, sex differences in (grand)offspring outcome (but not sex limitation) is a feature of the few human observational studies of transgenerational effects (Bygren et al., 2014; Miller et al., 2014; Northstone et al., 2014; Pembrey et al., 2006) and mammalian experiments showing transgenerational responses have reported numerous sex-specific effects after exposure during pregnancy (Dunn et al., 2011), or on paternal exposure before breeding. These can affect offspring of both sexes (Carone et al., 2010), solely/predominantly females (Ng et al., 2010), or solely/predominantly males (Drake and Walker, 2004; Franklin et al., 2010). There is growing experimental evidence in mammals that paternal transmission can be mediated by epigenetic inheritance in its broad sense, through sperm DNA methylation changes (Dias and Ressler, 2014) or altered sperm noncoding RNAs (Gapp et al., 2014).

Despite observational evidence of transgenerational responses in humans (Bygren et al., [Bibr b4],[Bibr b5]; Kaati et al., [Bibr b25],[Bibr b26]; Northstone et al., 2014; Pembrey et al., 2006), we know virtually nothing of the mediating molecular mechanisms in humans, so any attempt to interpret our findings in a mechanistic way is premature. A more appropriate approach may be to consider our findings in an evolutionary context. The evolutionary basis of contemporary phenotypic variation in human development and life history is an active research field (Kuzawa and Bragg, 2012). The “developmental origins of adult health and disease” hypothesis is largely confined to maternal nutrition and offspring growth and metabolic adaptations (Wells, 2011). There is, to our knowledge, no such theoretical treatment of observations on prenatal smoking effects.

A particular feature of our study is that we are able to compare transmission down the female and male lineages, and this raises another evolutionary aspect, namely the conflict that comes from sexually antagonistic traits. The two sexes are structurally and physiologically very different, with different life histories, yet they share the same genome (apart from the Y chromosome) on which evolutionary selection has to work. And because selection is based on transmission to offspring, sexually antagonistic theory involves both parent-of-origin issues as well as sexual dimorphism; and also of course mitochondria are only transmitted by the mother. These evolutionary conflicts lead to sex-specific adjustments to gene regulation both in terms of somatic gene expression and transmission through the germline. Genomic imprinting is widely considered an evolutionary consequence of a parental conflict in relation to fetal growth (Moore and Haig, 1991), with paternally expressed imprinted genes, e.g., IGF2 (Demetriou et al., 2014), favoring fetal growth and maternally expressed imprinted genes, e.g., PHLDA2 (Apostolidou et al., 2007; Ishida et al., 2012) suppressing fetal growth. However, as Table3 shows the only changes in fetal growth *per se* relate to the maternal line, where there are several routes for transmission of exposure-induced metabolic information that might explain the increased size at birth of the boys born to nonsmoking mothers who were themselves exposed in utero. Nevertheless evolutionary sexually antagonistic conflicts extend beyond just fetal growth and genomic imprinting. Frank and Crespi (2011) point out that “evolutionary conflicts cause opponents to push increasingly hard and in opposite directions on the regulation of traits. One can see only the intermediate outcome from the balance of the exaggerated and opposed forces.” However, these authors point out that a perturbation involving one side of the conflict can lead to pathology, such as misregulated growth. Smoking exposure might be just such a perturbation introducing an imbalance in the underlying (usually balanced) conflict that characterizes evolved human growth. With these evolved sex-specific child and adolescent growth patterns in mind, it is worth noting from Table3 that the boys have more increased lean mass (with or without increased muscle strength) with no change in height or fat mass, whilst the girls have changes in height, waist circumference, and fat mass, i.e., anthropometric features relevant to female reproductive success. These sex differences are in line with what sexual antagonistic theory would predict.

If the growth patterns we have observed can be replicated in further transgenerational smoking studies, the above evolutionary conceptualization might provide a suitable framework for research into molecular mechanisms. A starting point where there are sex differences in transmission and outcome are the sex chromosomes XY. It is worth noting that the transgenerational responses observed in the Överkalix study are compatible with X and Y segregation over three generations (Pembrey et al., [Bibr b37],[Bibr b36]) and pathology from evolutionary conflict suggests a theory of X chromosome versus autosome conflict over sexually antagonistic traits (Frank and Crespi, 2011). Furthermore, it has recently become clear that at least 150 circulating noncoding RNAs are encoded on the Y chromosome (Cortez et al., 2014). The adjustment in gene regulation that sexually antagonistic conflict theory requires may come from DNA based variation including repeats and mobile elements (Haig, 2012) working in conjunction with enhanced epigenetic responses to mediate enduring (often life-long) changes in gene expression. Whilst not entirely independent of the DNA sequence context, the epigenetic variation contributes to adaptation (Feinberg and Irizarry, 2010), and may mediate some parts of a transgenerational response. It is perhaps worth noting that maternal smoking has been shown to result in widespread differences in DNA methylation in cord blood samples (Joubert et al., 2012) and that the changes are male-specific at the differentially methylated region of the imprinted gene IGF2 (Murphy et al., 2012).

In conclusion, we believe maternal smoking is an important model for exploring transgenerational adaptive mechanisms. It is widespread permitting the possibility of replication studies and is of great public health importance in its own right.
