# Supplementary material for: The anthropometry of children and adolescents may be influenced by the prenatal smoking habits of their grandmothers: A longitudinal cohort study
Source: Am J Hum Biol. 2014 Aug 18;26(6):731–9. doi: 10.1002/ajhb.22594 (PMC4238812; doi:10.1002/ajhb.22594)
Supplement: Supplementary file 1 [file ajhb0026-0731-sd1.docx]

**S-Table 1.** PGM+M-. The adjusted relationships between the study child’s anthropological measures between ages 7 and 17 according to whether or not the paternal grandmother [PGM] smoked during the pregnancy resulting in the father: selecting only study children of mothers who did not smoke during their pregnancies [M-]. (significant relationships are printed in bold)

**Age at BOYS GIRLS**

**Measurement n_1:_n_2_ b [95% CI] P n_1:_n_2_ b [95% CI] P**

**Height [cm]**

7 846:1435 +0.23 [-0.22, +0.68] 0.309 879:1381 **+0.44 [-0.02, +0.90] 0.022**

9 776:1323 +0.32 [-0.22, +0.85] 0.250 860:1312 **+0.69 [+0.13, +1.25] 0.016**

11 747:1250 +0.38 [-0.26, +1.03] 0.245 805:1237 **+0.69 [+0.04, +1.34] 0.037**

13 648:1160 +0.71 [-0.14, +1.55] 0.100 720:1120 +0.53 [-0.06, +1.12] 0.076

15 552:964 +0.48 [-0.31, +1.28] 0.231 674:1019 +0.50 [-0.10, +1.09] 0.100

17 464:819 +0.46 [-0.30, +1.23] 0.237 647:1003 +0.57 [-0.05, +1.19] 0.071

**Weight [kg]**

7 846:1431 +0.25 [-0.11, +0.60] 0.173 879:1381 +0.36 [-0.02, +0.74] 0.064

9 780:1336 +0.32 [-0.30, +0.93] 0.311 868:1328 +0.58 [-0.05, +1.22] 0.071

11 748:1251 +0.51 [-0.37, +1.39] 0.254 805:1236 **+1.04 [+0.14, +1.94] 0.023**

13 648:1106 +1.05 [-0.08, +2.17] 0.067 719:1118 **+1.23 [+0.27, +2.20] 0.012**

15 551:963 **+ 1.67 [+0.38, +2.95] 0.011** 671:1016 **+1.18 [+0.14, +2.21] 0.026**

17 465:819 +1.48 [-0.02, +2.99] 0.053 647:1002 **+1.23 [+0.04, +2.42] 0.043**

**BMI**

7 846:1431 +0.09 [-0.06, +0.25] 0.242 879:1381 +0.11[-0.06, +0.29] 0.221

9 776:1323 +0.07 [-0.17, +0.30] 0.569 859:1312 +0.11 [-0.13, +0.36] 0.371

11 747:1250 +0.12 [-0.17, +0.41] 0.421 804:1236 +0.28 [-0.02, +0.59] 0.067

13 648:1106 +0.21 [-0.11, +0.52] 0.160 719:1118 **+0.35 [+0.03, +0.67] 0.032**

15 551:963 **+0.43 [+0.08, +0.78] 0.017** 671:1016 +0.32 [-0.03, +0.66] 0.069

17 464:819 +0.35 [-0.08, +0.78] 0.106 647:998 +0.31 [-0.08, +0.70] 0.121

**Waist Circumference [cm]**

7 845:1433 +0.22 [-0.19, +0.62] 0.299 879:1382 +0.32 [-0.11, +0.75] 0.140

9 778:1334 +0.16 [-0.50, +0.81] 0.642 868:1327 **+0.72 [+0.07, +1.37] 0.030**

11 748:1250 +0.27 [-0.59, +1.13] 0.533 806:1237 **+1.03 [+0.22, +1.83] 0.012**

13 646:1105 +0.46 [-0.44, +1.35] 0.318 718:1119 **+1.28 [+0.46, +2.09] 0.002**

15 450:777 +0.87 [-0.18, +1.92] 0.105 571:875 +0.89 [-0.04, +1.81] .060

**Fat Mass**

9 752:1264 -0.05 [-0.48, +0.38] 0.827 830:1264 +0.30 [-0.13, +0.73] 0.167

11 736:1227 +0.01 [-0.60, +0.61] 0.984 798:1217 +0.58 [-0.01, +1.17] 0.054

13 638:1088 +0.06 [-0.68, +0.79] 0.879 713:1102 **+0.75 [+0.07, +1.43] 0.030**

15 537:929 +0.66 [-0.25, +1.57] 0.153 639:983 +0.52 [-0.27, +1.31] 0.195

17 453:797 +0.17 [-0.97, +1.31] 0.773 626:970 +0.80 [-0.12, +1.71] 0.088

**Lean Mass**

9 752:1264 +0.24 [-0.03, +0.51] 0.078 830:1264 **+0.30 [+0.02, +0.57] 0.035**

11 736:1227 **+0.41 [+0.02, +0.80] 0.041** 798:1217 **+0.53 [+0.12, +0.93] 0.011**

13 638:1088 **+0.84 [+0.14, +1.53] 0.018** 713:1102 **+0.50 [+0.12, +0.88] 0.010**

15 537:929 **+1.00 [+0.30, +1.71] 0.005** 639:983 +0.31 [-0.09, +0.70] 0.129

17 453:797 **+1.36 [+0.63, +2.08] < 0.001** 626:970 +0.32 [-0.11, +0.75] 0.144

**Bone Mass**

9 752:1264 +0.02 [-0.00, +0.04] 0.071 830:1264 +0.02 [-0.00, +0.03] 0.099

11 736:1227 +0.02 [-0.00, +0.05] 0.082 798:1217 +0.03 [-0.00, +0.06] 0.064

13 **638:1088 +0.04 [+0.00, +0.09] 0.038** 713:1102 +0.03 [-0.01, +0.07] 0.099

15 **537:929 +0.06 [+0.01, +0.12] 0.016** 639:983 +0.02 [-0.02, +0.06] 0.223

17 **453:797 +0.08 [+0.03, +0.14] 0.004 626:970 +0.05 [+0.00, +0.09] 0.031**

n_1_ = no. where the paternal grandmother smoked in the pregnancy resulting in the birth of the study mother;

n_2_ = no. where the paternal grandmother did not smoke in the pregnancy resulting in the birth of the study mother.

Adjusted for parity, maternal education, housing tenure, and paternal smoking in pregnancy

**S-Table 2**. MGM+M- The adjusted relationships between the study child’s anthropological measures between ages 7 and 17 according to whether or not the maternal grandmother [MGM] smoked during the pregnancy resulting in the mother [M]: selecting only study children whose mothers who did not smoke during their pregnancies [M-] (significant relationships are printed in bold).

**Age at BOYS GIRLS**

**Measurement n_1:_n_2_ b [95% CI] P n_1:_n_2_ b [95% CI] P**

**Height [cm]**

7 663:1571 +0.03 [-0.46, +0.51] 0.915 669:1530 -0.06 [-0.56, +0.44] 0.816

9 623:1426 +0.06 [-0.52, +0.64] 0.851 655:1452 +0.26 [-0.34, +0.86] 0.392

11 592:1360 -0.02 [-0.72, +0.68] 0.950 609:1377 +0.22 [-0.49, +0.92] 0.547

13 504:1210 +0.29 [-0.63, +1.20] 0.538 550:1236 -0.15 [-0.78, +0.48] 0.643

15 447:1036 +0.36 [-0.48, +1.20] 0.404 502:1144 -0.16 [-0.79, +0.48] 0.622

17 359:898 +0.40 [-0.43, +1.24] 0.344 479:1121 -0.08 [-0.75, +0.59] 0.822

**Weight [kg]**

7 662:1568 +0.29 [-0.10, +0.67] 0.141 669:1530 +0.03 [-0.38, +0.44] 0.893

9 628:1437 +0.61 [-0.05, +1.26] 0.069 661:1468 +0.46 [-0.22, +1.14] 0.181

11 593:1361 **+0.48 [+0.08, +1.94] 0.033** 608:1377 +0.46 [-0.51, +1.43] 0.354

13 504:1210 +0.89 [-0.33, +2.11] 0.154 549:1234 +0.20 [-0.84, +1.23] 0.709

15 446:1035 **+1.50 [+0.14, +2.86] 0.030** 500:1140 +0.51 [-0.60, +1.62] 0.368

17 359:899 **+2.09 [+0.47, +3.71] 0.012** 479:1120 +0.65 [-0.64, +1.93] 0.323

**BMI**

7 662:1568 **+0.16 [+0.00, +0.33] 0.049** 669:1530 +0.03 [-0.16, +0.22] 0.774

9 623:1426 **+0.28 [+0.03, +0.53] 0.028** 655:1451 +0.18 [-0.08, +0.44] 0.179

11 592:1360 **+0.41 [+0.11, +0.72] 0.009** 608:1376 +0.14 [-0.18, +0.47] 0.394

13 504:1210 +0.24 [-0.11, +0.58] 0.175 549:1234 +0.10 [-0.24, +0.45] 0.554

15 446:1035 **+0.42 [+0.05, +0.79] 0.028** 500:1140 +0.22 [-0.15, +0.58] 0.244

17 359:898 **+0.55 [+0.09, +1.01] 0.020** 476:1119 +0.17 [-0.25, +0.59] 0.425

**Waist Circumference [cm]**

7 662:1569 **+0.46 [+0.03, +0.90] 0.037** 669:1530 +0.18 [-0.28, +0.64] 0.447

9 626:1436 +0.61 [-0.09, +1.30] 0.087 660:1468 +0.64 [-0.06, +1.33] 0.072

11 593:1360 **+1.10 [+0.18, +2.01] 0.019** 610:1377 +0.57 [-0.29, +1.43] 0.192

13 502:1209 +0.33 [-0.64, +1.30] 0.503 549:1234 +0.44 [-0.43, +1.32] 0.322

15 365:837 +**1.12 [+0.01, +2.22] 0.049** 435:972 +0.62 [-0.36, +1.60] 0.217

**Fat Mass**

9 593:1370 +0.31 [-0.15, +0.77] 0.190 632:1404 +0.34 [-0.12, +0.80] 0.151

11 583:1335 +0.63 [-0.15, +1.27] 0.055 600:1360 +0.13 [-0.51, +0.76] 0.697

13 498:1188 +0.10 [-0.70, +0.90] 0.807 540:1222 +0.01 [-0.72, +0.73] 0.981

15 426:1012 +0.36 [-0.61, +1.32] 0.466 481:1096 +0.30 [-0.55, +1.14] 0.491

17 353:871 +0.59 [-0.64, +1.82] 0.344 465:1084 +0.33 [-0.65, +1.31] 0.509

**Lean Mass**

9 593:1370 +0.27 [-0.02, +0.56] 0.063 632:1404 +0.15 [-0.14, +0.45] 0.303

11 583:1335 +0.40 [-0.02, +0.82] 0.062 600:1360 +0.21 [-0.23, +0.64] 0.355

13 498:1188 +0.71 [-0.04, +1.47] 0.064 540:1222 -0.06 [-0.47, +0.35] 0.763

15 426:1012 **+0.86 [+0.11, +1.62] 0.026** 481:1096 +0.08 [-0.34, +0.51] 0.699

17 353:871 **+1.49 [+0.70, +2.28] <0.001** 465:1084 +0.20 [-0.26, +0.66] 0.396

**Bone Mass**

9 593:1370 +0.01 [-0.01, +0.03] 0.183 632:1404 +0.01 [-0.01, +0.03] 0.335

11 583:1335 +0.02 [-0.01, +0.05] 0.116 600:1360 +0.01 [-0.03, +0.04] 0.730

13 498:1188 +0.04 [-0.01, +0.08] 0.112 540:1222 -0.01 [-0.04, +0.03] 0.788

15 426:1012 +0. 04 [-0.01, +0.10] 0.132 481:1096 +0.01 [-0.03, +0.05] 0.682

17 **353:871 +0.07 [+0.01, +0.14] 0.021** 465:1084 +0.02 [-0.03, +0.06] 0.483

n_1_ = no. where the maternal grandmother smoked in the pregnancy resulting in the birth of the study mother;

n_2_ = no. where the maternal grandmother did not smoke in the pregnancy resulting in the birth of the study mother.

Adjusted for parity, maternal education, housing tenure, and paternal smoking in pregnancy

**S-Table 3**. PGM+M+ The adjusted relationships between the study child’s anthropological measures between ages 7 and 17 according to whether or not the paternal grandmother [PGM] smoked during the pregnancy resulting in the father: selecting only study children of mothers who did smoke during their pregnancies [M+] (significant relationships are printed in bold).

**Age at BOYS GIRLS**

**Measurement n_1:_n_2_ b [95% CI] P n_1:_n_2_ b [95% CI] P**

**Height [cm]**

7 264:279 -0.06 [-0.98, +0.87] 0.900 256:246 -0.14 [-1.08, +0.79] 0.763

9 235:242 -0.11 [-1.23, +1.00] 0.841 228:226 -0.61 [-1.76, +0.54] 0.299

11 207:214 +0.74 [-0.65, +2.13] 0.295 215:211 -0.74 [-2.14, +0.66] 0.301

13 165:188 +0.83 [-1.13, +2.78] 0.407 152:172 +0.71 [-0.67, +2.08] 0.312

15 126:156 +0.47 [-1.45, +2.39] 0.630 154:163 +0.68 [-0.65, +2.01] 0.316

17 108:131 +0.73 [-1.19, +2.65] 0.457 145:155 +0.87 [-0.48, +2.22] 0.205

**Weight [kg]**

7 263:278 +0.13 [-0.62, +0.87] 0.739 256:246 -0.16 [-1.02, +0.70] 0.708

9 238:243 -0.18 [-1.50, +1.15] 0.793 229:230 -1.03 [-2.57, +0.51] 0.188

11 207:215 +0.89 [-1.01, +2.80] 0.357 216:210 -0.24 [-2.31, +1.83] 0.819

13 165:188 +0.75 [-1.84, +3.33] 0.570 150:172 +0.76 [-1.94, +3.45] 0.580

15 126:156 -1.06 [-3.82, +1.72] 0.454 154:163 +0.69 [-1.99, +3.38] 0.611

17 109:131 -1.70 [-5.32, +1.92] 0.355 145:154 +2.17 [-0.98, +5.32] 0.176

**BMI**

7 263:278 +0.10 [-0.24, +0.44] 0.560 256:246 -0.08 [-0.49, +0.33] 0.696

9 235:241 -0.01 [-0.54, +0.52] 0.972 228:226 -0.37 [-0.97, +0.24] 0.236

11 207:214 +0.17 [-0.49, +0.83] 0.607 215:210 +0.07 [-0.64, +0.79] 0.844

13 165:188 +0.08 [-0.65, +0.81] 0.832 150:172 +0.12 [-0.77, +1.00] 0.798

15 126:156 -0.39 [-1.16, +0.37] 0.315 154:163 +0.06 [-0.86, +0.99] 0.893

17 108:129 -0.54 [-1.52, +0.44] 0.281 145:154 +0.59 [-0.51, +1.68] 0.292

**Waist Circumference [cm]**

7 263:278 +0.19 [-0.65, +1.03] 0.651 256:246 +0.04 [-0.98, +1.06] 0.936

9 238:244 +0.17 [-1.27, +1.61] 0.814 229:229 -1.10 [-2.65, +0.45] 0.165

11 207:214 +0.66 [-1.27, +2.60] 0.502 215:210 +0.15 [-1.70, +1.99] 0.874

13 165:187 +0.60 [-1.44, +2.64] 0.564 152:171 +0.94 [-1.37, +3.25] 0.424

15 103:121 -1.08 [-3.36, +1.21] 0.356 128:138 +**2.49 [+0.15, +4.83] 0.037**

**Fat Mass**

9 227:235 -0.26 [-1.17, +0.66] 0.580 212:221 -0.32 [-1.33, +0.69] 0.532

11 203:210 +0.46 [-0.74, +1.65] 0.450 208:208 +0.20 [-1.20, +1.60] 0.776

13 159:185 -0.02 [-1.66, +1.62] 0.982 147:168 +0.64 [-1.31, +2.59] 0.519

15 113:147 -0.38 [-3.38, +0.62] 0.175 140:150 +0.42 [-1.65, +2.50] 0.688

17 105:126 -0.79 [-3.38, +1.79] 0.546 140:149 +1.32 [-1.09, +3.72] 0.283

**Lean Mass**

9 227:235 -0.22 [-0.80, +0.35] 0.444 212:221 -0.44 [-1.02, +0.15] 0.144

11 203:210 +0.31 [-0.54, +1.15] 0.474 208:208 -0.45 [-1.33, +0.44] 0.322

13 159:185 +0.50 [-1.12, +2.11] 0.546 147:168 +0.45 [-0.49, +1.39] 0.346

15 113:147 +0.37 [-1.43, +2.18] 0.684 140:150 +0.37 [-0.58, +1.32] 0.444

17 105:126 +0.11 [-1.57, +1.79] 0.897 140:149 +0.56 [-0.48, +1.60] 0.292

**Bone Mass**

9 227:235 -0.01 [-0.05, +0.02] 0.442 212:221 -0.02 [-0.05, +0.03] 0.466

11 203:210 +0.03 [-0.02, +0.08] 0.235 208:208 -0.01 [-0.07, +0.05] 0.764

13 159:185 +0.04 [-0.06, +0.14] 0.430 147:168 +0.06 [-0.03, +0.15] 0.180

15 113:147 +0.00 [-0.13, +0.14] 0.955 140:150 +0.07 [-0.02, +0.16] 0.147

17 105:126 +0.02 [-0.12, +0.16] 0.764 140:149 +0.08 [-0.02, +0.17] 0.116

n_1_ = no. where the paternal grandmother smoked in the pregnancy resulting in the birth of the study father;

n_2_ = no. where the paternal grandmother did not smoke in the pregnancy resulting in the birth of the study father.

Adjusted for parity, maternal education, housing tenure, and paternal smoking in pregnancy

**S-Table 4.** MGM+M+ The adjusted relationships between the study child’s anthropological measures between ages 7 and 17 according to whether or not the maternal grandmother [MGM] smoked during the pregnancy resulting in the mother: selecting only study children of mothers who did smoke during their pregnancies [M+] (significant relationships are printed in bold).

**Age at BOYS GIRLS**

**Measurement n_1:_n_2_ b [95% CI] P n_1:_n_2_ b [95% CI] P**

**Height [cm]**

7 246:290 +0.44 [-0.50, +1.38] 0.354 211:280 -0.90 [-1.85, +0.05] 0.062

9 205:272 +0.51 [-0.61, +1.64] 0.369 189:257 -**1.83 [-3.00, -0.67] 0.002**

11 183:239 +0.12 [-1.30, +1.54] 0.868 173:242 -1.09 [-2.53, +0.36] 0.139

13 153:198 +0.33 [-1.68, +2.33] 0.750 134:184 -1.06 [-2.47, +0.34] 0.138

15 124:153 +0.29 [-1.65, +2.23] 0.769 135:180 -0.38 [-1.74, +0.98] 0.581

17 113:123 +0.81 [-1.15, +2.77] 0.418 138:161 -0.71 [-2.06, +0.65] 0.305

**Weight [kg]**

7 245:289 +0.57 [-0.19, +1.33] 0.143 211:280  **-0.90 [-1.78, -0.03] 0.043**

9 207:274 +0.60 [-0.75, +1.96] 0.380 192:259 **-2.24 [-3.81, -0.67] 0.005**

11 184:239 -0.07 [-2.03, +1.89] 0.944 173:242 -1.83 [-3.97, +0.31] 0.094

13 153:198 +0.37 [-2.29, +3.03] 0.783 134:182 -1.73 [-4.49, +1.04] 0.221

15 124:153 -0.26 [-3.12, +2.60] 0.857 135:180 -1.77 [-4.49, +0.95] 0.201

17 113:124 -1.39 [-5.31, +2.54] 0.486 137:161 -1.09 [-4.27, +2.08] 0.499

**BMI**

7 245:289 +0.24 [-0.11, +0.59] 0.184 211:280 -0.31 [-0.73, +0.10] 0.142

9 205:271 +0.11 [-0.43, +0.65] 0.686 189:257 -0.55 [-1.17, +0.07] 0.084

11 183:239 -0.07 [-0.75, +0.62] 0.849 173:241 -0.45 [-1.19, +0.29] 0.234

13 153:198 +0.03 [-0.73, +0.79] 0.941 134:182 -0.31 [-1.23, +0.60] 0.503

15 124:153 -0.17 [-0.96, +0.63] 0.680 135:180 -0.59 [-1.52, +0.35] 0.219

17 113:121 -0.56 [-1.63, +0.52] 0.308 137:161 -0.17 [-1.28, +0.94] 0.763

**Waist Circumference [cm]**

7 245:289 +0.72 [-0.14, +1.58] 0.102 211:280 -0.63 [-1.67, +0.41] 0.231

9 207:275 +0.34 [-1.13, +1.81] 0.646 191:259 **-1.67 [-3.27, -0.08] 0.040**

11 184:238 -0.12 [-2.11, +1.88] 0.908 172:242 -1.51 [-3.42, +0.40] 0.121

13 153:197 +0.45 [-1.65, +2.55] 0.674 133:184 -1.09 [-3.48, +1.30] 0.370

15 99:120 -0.93 [-3.30, +1.44] 0.440 114:153 +0.09 [-2.30, +2.48] 0.939

**Fat Mass**

9 196:265 +0.15 [-0.80, +1.10] 0.753 179:246 -1.00 [-2.04, +0.04] 0.058

11 182:232 -0.35 [-1.60, +0.90] 0.581 166:240 -1.01 [-2.46, +0.44] 0.171

13 149:193 -0.53 [-2.26, +1.19] 0.545 131:178 -0.60 [-2.61, +1.42] 0.561

15 115:140 -1.12 [-3.23, +1.00] 0.299 121:165 -1.12 [-3.25, +1.00] 0.299

17 108:120 -1.32 [-4.19, +1.55] 0.365 135:154 -0.31 [-2.72, +2.10] 0.803

**Lean Mass**

9 196:265 +0.46 [-0.12, +1.04] 0.119 179:246 **-0.79 [-1.38, -0.20] 0.009**

11 182:232 +0.18 [-0.68, +1.03] 0.683 166:240 -0.69 [-1.60, +0.21] 0.134

13 149:193 +0.40 [-1.25, +2.06] 0.632 131:178 -0.48 [-1.46, +0.50] 0.333

15 115:140 +0.91 [-0.92, +2.74] 0.328 121:165 -0.76 [-1.73, +0.22] 0.130

17 108:120 +0.35 [-1.40, +2.10] 0.692 135:154 -0.06 [-1.11, +0.98] 0.904

**Bone Mass**

9 196:265 +0.02 [-0.02, +0.06] 0.285 179:246 -0.04 [-0.08, +0.00] 0.058^a^

11 182:232 +0.02 [-0.04, +0.07] 0.583 166:240 -0.04 [-0.11, +0.02] 0.184

13 149:193 +0.02 [-0.09, +0.12] 0.762 131:178 -0.04 [-0.13, +0.06] 0.425

15 115:140 +0.04 [-0.09, +0.18] 0.537 121:165 -0.06 [-0.15, +0.04] 0.225

17 108:120 -0.01 [-0.15, +0.13] 0.909 135:154 -0.02 [-0.12, +0.07] 0.632

^a^P = 0.045 interaction

n_1_ = no. where the maternal grandmother smoked in the pregnancy resulting in the birth of the study mother;

n_2_ = no. where the maternal grandmother did not smoke in the pregnancy resulting in the birth of the study mother.

Adjusted for parity, maternal education, housing tenure, and paternal smoking in pregnancy

**S-Table 5.** Adjusted associations of mean grip strength at age 11 and fitness at age 11 according to prenatal smoking of mother and grandparents (significant relationships are printed in bold).

**Group BOYS GIRLS**

**Comparisons n_1:_n_2_ b [95% CI] P n_1:_n_2_ b [95% CI] P**

**Grip Strength**

**PGM+M-** 704:1172 +0.35[-0.03, +0.73] 0.067 751:1176 +0.21[-0.14, +0.57] 0.241

MGM+M- 562:1268 **+0.52[+0.11, +0.92] 0.012** 577:1293 -0.18[-0.56, +0.20] 0.345

PGM+M+ 197:197 +0.43[-0.43, +1.29] 0.330 201:193 -0.57[-1.39, +0.24] 0.167

MGM+M+ 171:222 +0.58[-0.31, +1.47] 0.202 155:230 -0.60[-1.45, +0.26] 0.169

**Fitness**

PGM+M- 324:598 -0.70[-1.20, +1.06] 0.903 509:769 +0.58[-0.45, +1.61] 0.270

MGM+M- 275:626 **+2.08[+0.91, +3.26] 0.001** 378:861 +0.38[-0.74, +1.50] 0.503

PGM+M+ 102:104 +0.92[-1.43, +3.27] 0.443 124:120 -0.31[-2.75, +2.12] 0.800

MGM+M+ 90:114 +1.20[-1.16, +3.57] 0.318 107:131 -0.38[-2.83, +2.08] 0.762

MGM = maternal grandmother; PGM = paternal grandmother; M = mother; + = smoked prenatally; - = did not smoke prenatally.
